# New Technologies for Blood Glucose Management in Elderly Diabetics: An Interpretation of the *Guidelines for the Management of Diabetes Mellitus in the Elderly in China* (2024 Edition)

**DOI:** 10.1002/agm2.70044

**Published:** 2025-10-10

**Authors:** Qingyun Cai, Peiyi Zhao, Xinda Chen, Shuyi Yu, Lixin Guo, Qi Pan

**Affiliations:** ^1^ Department of Endocrinology Beijing Hospital Beijing China; ^2^ Graduate School of Peking Union Medical College Chinese Academy of Medical Sciences Beijing China

**Keywords:** elderly diabetes, glycemic management, guidelines interpretation, insulin injection, technological advances

## Abstract

The burden of diabetes in the elderly is becoming increasingly severe due to the aging population. Elderly patients with diabetes face unique challenges due to multiple comorbidities, atypical symptoms, an increased risk of hypoglycemia, and limited self‐management capabilities. The *Guidelines for the Management of Diabetes Mellitus in the Elderly in China* (2024 Edition) advocate for a stratified, individualized approach that addresses the heterogeneous pathophysiologic profiles of these patients. Recent technological advances, including continuous glucose monitoring, smart insulin pens, needle‐free injection systems, and new insulin pumps, have significantly improved glycemic management. The updated guidelines systematically review evidence‐based recommendations for managing diabetes in the elderly, emphasizing the benefits of modern monitoring and insulin delivery technologies. This article interprets the updated guidelines with a focus on the technical principles, clinical benefits, and application dilemmas to ultimately improve long‐term outcomes among elderly patients with diabetes.

## Graphical abstract

This article reviews the latest advances in diabetes management technologies, and expound the technical principles, clinical benefits, and application dilemmas for elderly diabetes patients.

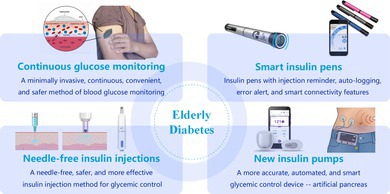

## 1 Introduction

Elderly diabetic patients are defined as individuals > 65 years of age, including patients diagnosed with diabetes before and after reaching this age [1]. Compared to the general adult diabetic patient population, elderly diabetic patients often present with multiple complications and comorbidities, atypical symptoms, a high risk of hypoglycemia, and poor self‐management capabilities [2, 3, 4, 5, 6, 7]. Therefore, there is an urgent need to establish an individualized management strategy for glucose target setting, drug selection, and the choice of technical adjunctive therapies based on the heterogeneity of the pathophysiologic characteristics [8].

Significant advances have been made in diabetes monitoring and insulin delivery technologies in recent years that have substantially optimized blood glucose management for elderly patients with diabetes. The *Guidelines for the Management of Diabetes Mellitus in the Elderly in China* (2024 Edition) systematically elucidate the latest evidence‐based recommendations and clinical applications of technological advances [8]. This review delves into the key updates of the guidelines, expounding the technical principles, clinical benefits, and application dilemmas from three dimensions to provide scientific guidance for the use of new technologies that enhance comprehensive management and improve long‐term outcomes.

## 2 Continuous Glucose Monitoring (CGM)

Capillary blood glucose monitoring via fingertip sampling is a commonly used method for daily blood glucose measurement [9]. Increasing the frequency of testing can reduce the risk of hypoglycemia. However, capillary blood glucose monitoring has some limitations: frequent pricking may increase anxiety, fear, or frustration; and irregularities at the sampling sites, such as poor blood supply to the fingertips or skin sclerosis, may result in inaccurate readings [10, 11, 12]. Moreover, because blood sampling is intermittent, fingertip capillary glucose monitoring only provides a snapshot of the glucose concentration. Even with frequent checks, episodes of hyperglycemia and hypoglycemia may be missed [13].

CGM represents advanced, minimally invasive glucose monitoring technology. CGM utilizes a subcutaneously implanted microelectrode to measure glucose levels in the interstitial fluid and converts these measurements into electrical signals for continuous tracking throughout the day [14]. Unlike traditional fingertip glucose monitoring, CGM typically lasts between 7 and 14 days, while some models extend up to 180 days and automatically record glucose levels at intervals ranging from 1 to 15 min [15]. The system stores and generates a comprehensive glucose profile. Then, real‐time glucose readings are transmitted to a smartphone or other device via Bluetooth for convenient access [16].

CGM has been shown to effectively improve hemoglobin A1c levels and reduce daily glucose variability [17, 18, 19]. Many studies focusing on elderly diabetic patients have consistently demonstrated the benefits of CGM in lowering blood glucose levels without increasing the risk of hypoglycemia [20, 21, 22, 23, 24]. Elderly diabetic patients often exhibit diminished residual β‐cell function, pronounced glucose fluctuations, and impaired ability to respond to hypoglycemia [25, 26]. The WISDM trial showed that CGM significantly improves hypoglycemia awareness and reduces the occurrence of severe hypoglycemic events by up to 90% [27]. This secondary analysis of the WISDM study involving 203 elderly patients with type 1 diabetes in the United States demonstrated limitations in terms of race and educational background (93% are Caucasians, and 94% have received college education or above).

Additionally, another US‐based research involving elderly diabetic patients revealed that CGM use leads to an increase in time spent in the target glucose range (TIR) with outcomes comparable to those observed in younger populations [28]. CGM also significantly reduces the need for frequent fingerstick testing, alleviating patient discomfort and enhancing adherence to glucose management protocols [29]. Reducing the frequency of fingerstick testing is particularly beneficial for elderly individuals with cognitive, behavioral, or visual impairment [30]. Notably, current evidence lacks robust studies specifically investigating CGM applications in China's elderly diabetic population.

CGM has been included in China's national medical insurance reimbursement catalog, establishing it as an excellent glucose monitoring option for elderly diabetic patients. However, CGM utilization rates remain at only 28.2% among individuals aged ≥ 80 years, significantly lower than the 75.3% observed in younger populations [31]. The implementation of CGM in geriatric care faces two primary challenges: First, the significant heterogeneity within the elderly population leads to variations in applying the technology. The wearing and replacement of sensors require technical skill, and some elderly patients, due to cognitive impairment or reduced fine motor skills, may be unable to independently operate the sensors. Second, the psychological burden associated with the technology requires particular attention. Frequent alarm notifications may cause discomfort, especially when nighttime alarms interrupt sleep, potentially leading to anxiety, depression, and other adverse psychological effects. Strict blood glucose thresholds may not be suitable for elderly individuals. Instead, glucose limits that align with the patient's age and health status should be considered [8].

## 3 Smart Insulin Pens

The insulin pen, invented in 1985, houses insulin in a cartridge and delivers insulin subcutaneously through a syringe [32]. The insulin pen quickly became an integral part of multiple daily injection (MDI) therapy and, for some patients, standardized insulin injection is key to achieving blood glucose control goals [33]. However, only 67.53% of elderly diabetic patients demonstrated good insulin adherence, which is still lower compared to other chronic diseases, such as hypertension (80%) [34].

The insulin pen has evolved from a simple syringe into a more sophisticated device, incorporating a screen display and a button‐operated injection mechanism that replaced the traditional press‐to‐inject method. Smart insulin pens offer automatic recording and intelligent connectivity features, accurately tracking hundreds of insulin doses, including the date, time, and dosage of the last injection, insulin type, and temperature range [35]. This transformation of insulin injection behavior into “digital” data reduces the burden of manual recordkeeping and improves accuracy. Additionally, smart insulin pens feature visual and auditory alerts to prevent missed or repeated injections and ensure accurate timing and dosage [36]. The smart insulin pen can connect with smart devices supporting near‐field communication technology, transferring insulin injection records to a smartphone, thus providing a “visual” representation of injection behavior [37].

Elderly diabetic patients often face challenges, such as memory loss, impaired vision, and difficulty with hand operations, making daily insulin injections more difficult [38, 39]. The memory function, user‐friendly interface, and intelligent sharing services of the smart insulin pen significantly reduce these barriers [40, 41]. The alert function contributes to reminding elderly patients to accurately inject insulin, reducing the risk of missed, repeated, or incorrect injections. The smart insulin pen has been demonstrated to reduce the incidence of missed insulin doses by 43%, while simultaneously extending the glucose time in range (TIR) by 8.5% in comparison with the baseline period. Additionally, significant reductions in glucose time below range (TBR) and glucose time above range (TAR) have been observed. Notably, elderly patients appear to derive greater benefits from the use of this technology compared to younger individuals [42, 43]. In real‐world studies, the average TIR in type 1 diabetes mellitus (T1DM) patients using the smart insulin pen was 59%, which was higher than that of patients using CGM combined with a traditional insulin pump (53.3%) [44]. The use of smart insulin pens is projected to improve clinical outcomes at lower costs compared to standard care [45]. This finding highlights the superiority of the smart insulin pen in daily blood glucose management, although it is based on adult patients with T1DM.

Current pricing for smart insulin pens ranges ¥100–300, with partial medical insurance coverage available in certain regions, substantially alleviating financial burdens. However, two critical limitations hinder widespread adoption among elderly patients. Some elderly patients may have difficulty with the physical manipulation of the pen, such as properly inserting the needle or replacing the insulin cartridge. Moreover, reliance on smartphones for data synchronization may present barriers for elderly individuals who are unfamiliar with mobile devices or have difficulty managing mobile devices.

## 4 Needle‐Free Insulin Injections

Insulin has a crucial role in diabetes treatment, but irregular injection behaviors are prevalent among elderly diabetic patients [46, 47]. Inconsistent rotation of injection sites, reuse of needles, and incorrect injection techniques may result in various injection‐related complications, including but not limited to bleeding at the injection site, pain, and lipohypertrophy [48, 49, 50]. Furthermore, psychological factors, including fear of needles and concerns about injection pain, may cause patients to decline insulin therapy or deliberately reduce the frequency and dosage of injections [51, 52].

The introduction of needle‐free injectors has significantly addressed many challenges faced by elderly diabetic patients. Needle‐free insulin injection utilizes a pressure source, such as gas, electromagnetic force, or springs, to generate a brief high‐pressure burst that propels insulin through a fine nozzle, creating a high‐velocity jet that rapidly permeates the skin and diffuses into the subcutaneous tissue, leading to the quick onset of action [53]. This jet travels at speeds of 200–220 m/s and penetrates the soft tissue to a limited depth (4–6 mm), minimizing stimulation to the subcutaneous tissue and nerve endings [54]. This delivery method reduces the anxiety associated with traditional needle injections, alleviates injection pain, and lowers the risk of adverse reactions related to needle use, ultimately improving patient adherence to treatment [55].

Needle‐free insulin injection has become a recommended method for insulin administration in the *Guidelines for the Management of Diabetes Mellitus in the Elderly in China* (2024 Edition) [8]. Compared to traditional needle injections, needle‐free insulin injection has been shown to effectively control fasting glucose levels and significantly reduce 24‐h average glucose levels, peak glucose levels, and fasting glucose variability [56, 57]. Needle‐free injection also allows for faster absorption and a shorter time to peak concentration, resulting in an earlier peak in the glucose infusion rate [58]. Needle‐free injection improves early postprandial glucose control, especially in the first hour after dinner, which may specifically benefit elderly patients at risk of immediate postprandial hyperglycemia and late postprandial hypoglycemia [59]. In addition, a needle‐free injector significantly reduces the required insulin dosage for glycemic control, with the reduction directly proportional to the dose administered [60]. Notably, variations in skin thickness do not affect insulin absorption when using needle‐free injection [61]. The absence of a needle in the injection process greatly improves the treatment experience [62]. Based on these advantages, needle‐free injection has been recommended as one of the insulin delivery methods in the *Chinese Diabetes Injection Guidelines* [63].

As a new technology, needle‐free injection lacks large‐scale clinical studies specifically focused on elderly diabetic patients. Existing studies have been limited to small cohorts with maximum follow‐up durations of 18 months, leaving the technology's effects on chronic complications and long‐term outcomes in geriatric patients undetermined. Compared to insulin pen injections, the operation of needle‐free injectors is more complex and requires professional guidance. Patients must master the technique before they can independently administer injections. Moreover, the significantly higher cost of needle‐free injectors compared to insulin pens, and the condition that currently only a limited number of consumables are covered by medical insurance, increase the financial burden on elderly patients, especially for those in less economically developed areas.

## 5 New Insulin Pumps

The insulin pump continuously administers small doses of insulin in a controlled manner in alignment with physiologic secretion patterns and tailored to the patient's daily activities and dietary habits [64]. Insulin pumps have evolved into AI‐controlled continuous subcutaneous insulin infusion (CSII) devices. The safety and efficacy of insulin pump therapy in elderly patients have been demonstrated by several studies [65, 66, 67, 68]. Importantly, CSII is significantly effective in reducing the incidence and progression of retinopathy and proteinuria, as well as fatal cardiovascular risk and all‐cause mortality among elderly patients with diabetes [69, 70, 71].

Based on the traditional insulin pumps, the new insulin pump (sensor augmented pump [SAP]) incorporates CGM, hyperglycemia, and hypoglycemia threshold alarms and trend prediction capabilities [72]. This advanced insulin pump achieved a significantly greater HbA1c percentage and glucose variability reduction with less insulin dose compared to MDIs [73, 74, 75]. Elderly diabetic patients are at a heightened risk for severe hypoglycemia, and insulin pumps significantly enhance the safety of insulin therapy [76]. According to the ORACL study, elderly patients using SAP experienced a substantial reduction in the risk of hypoglycemia, particularly during the night, compared to patients utilizing CGM in conjunction with MDIs [77].

Hybrid closed‐loop (HCL) systems further facilitate blood glucose management for elderly diabetic patients. An HCL system continuously monitors glucose levels and intelligently calculates the necessary insulin dose based on real‐time glucose readings. Then, an HCL system automatically delivers insulin, facilitating closed‐loop glucose management and earning the nickname “artificial pancreas” [78]. An HCL system significantly reduces the burden associated with manual blood glucose testing, data interpretation, calculation, and injection. Therefore, the psychological, cognitive, and functional limitations commonly observed in the elderly population are addressed by an HCL system. A multicenter randomized controlled trial involving 168 patients with T1DM demonstrated that the HCL group achieved better glycemic control and a lower risk of hypoglycemia compared to the SAP group [79]. Additionally, a meta‐analysis of 40 early outpatient studies reported that an HCL system not only shortened the time spent in the TIR but also reduced hypoglycemia in patients with T1DM [80].

Users of the HCL system have reported psychological benefits, including reduced anxiety, improved sleep, enhanced safety due to better overnight glucose control, and less restrictive eating habits [81]. The updated HCL system incorporates meal detection technology, automatically collecting physiologic data after meals and estimating dietary composition, enabling the delivery of the appropriate insulin dose for glucose correction [82]. Additionally, a new generation of tubeless insulin pumps has been developed [83]. It should be noted that the generalizability of these findings to elderly Chinese patients with T2DM remains uncertain, as clinical studies specifically targeting this population are currently lacking.

Although an HCL system offers significant advantages in glycemic management for elderly patients with diabetes, several technical and economic limitations remain. First, the physiologic heterogeneity of older adults poses challenges for algorithm adaptability. Reduced insulin sensitivity, impaired postprandial glucagon regulation, and declining hepatic and renal function may lead to inaccurate insulin dose recommendations, increasing the risk of cumulative hypoglycemia. Second, cognitive decline, impaired vision, reduced manual dexterity, lack of caregiver support, and insufficient knowledge of insulin pump operation may limit the feasibility and adherence to HCL therapy. Furthermore, clinical studies specifically evaluating an HCL system in elderly populations are limited, necessitating more robust evidence to confirm the long‐term efficacy and safety. Finally, the high economic cost of HCL systems remains a barrier to widespread adoption, especially for patients with constrained healthcare resources.

## 6 Conclusion

With the accelerated aging of the population in China, the prevalence of diabetes among the elderly has risen to the highest globally and continues to increase. Elderly patients often exhibit limited disease awareness, poor self‐management abilities, and suboptimal treatment adherence, which significantly hinder effective glycemic control. Based on the evidence‐based recommendations of the *Guidelines for the Management of Diabetes Mellitus in the Elderly in China* (2024 Edition), this review highlights the latest advances in diabetes management technologies, emphasizing the role of digital and intelligent solutions in optimizing blood glucose control, improving treatment adherence, and enhancing the quality of life.

From CGM and smart insulin pens to needle‐free injection technologies and HCL systems, these innovations not only reduce the risk of hypoglycemia and improve insulin therapy precision but also facilitate the transition from patient‐dependent management to data‐driven, automated adjustments, offering more individualized solutions for elderly diabetic patients. However, due to the unique challenges faced by the elderly, including limited technological proficiency, psychological issues, and health literacy, the clinical implementation of these new blood glucose management technologies faces multiple practical barriers.

It is recommended that the utilization of novel technologies in elderly patients with diabetes should adhere to the “STEPS” principles (Safety, Tolerance, Ease‐of‐use, Personalization, Sustainability). Individualized decision‐making recommendations are stratified for each elderly diabetic patient based on the available evidence and clinical experience. For instance, stepwise selection based on cognitive function: The HCL system combined with CGM is recommended for elderly patients with normal cognitive function, and the smart insulin pen combined with CGM or traditional glucose meter is recommended for patients with mild‐to‐moderate cognitive impairment, in order to avoid the risks associated with complex operations. Meanwhile, in terms of the economic‐benefit balance strategy, a “three‐tiered pyramid” scheme is adopted, with traditional insulin pens combined with CGM recommended for the basic tier (with lower disposable healthcare costs), a single new technology recommended for the intermediate tier, and HCL/needle‐free injections combined with CGM for the advanced tier, provided that long‐term adherence is assessed. The implementation of novel technologies should be embedded in a comprehensive geriatric assessment framework and be dynamically adjusted by obtaining feedback on its use through short‐ and long‐term follow‐up.

Technological advances in blood glucose management will extend beyond breakthroughs in algorithms or hardware in the future, focusing more on addressing the digital health barriers for the elderly population. Technological advances are crucial to overcoming the challenges of technological complexity and the adaptation between these technologies and the operational capabilities of elderly users. Additionally, a study has confirmed the compliance of elderly patients in using CGM can be significantly improved through targeted education [84]. The establishment of a “technology‐humanities” dual‐track training system in healthcare institutions contributes to improving the technical guidance skills of healthcare providers and the digital health literacy of elderly patients, which will foster a positive cycle of “technology usability—patient acceptance—clinical benefits”. Only with the collaborative support of technological innovation, institutional guarantees, and human‐centered care can elderly patients with diabetes truly benefit from advances in blood glucose management technologies, ultimately achieving the goal of healthy aging.

## Author Contributions

Q.C. collected the data and drafted the article. P.Z. collected the data. X.C. collated the data. S.Y. contributed to the discussion. L.G. reviewed and edited the manuscript. Q.P. revised the article and approved the submitted version. All authors approved the final version of the manuscript.

## Conflicts of Interest

The authors declare no conflicts of interest.
